# Epidural Effusion as Allergic Reaction Following Polyetheretherketone Cranioplasty: An Illustrative Case and Review of the Literature

**DOI:** 10.7759/cureus.21390

**Published:** 2022-01-18

**Authors:** Lisa B Shields, Meena Vessell, Ian S Mutchnick

**Affiliations:** 1 Neurological Surgery, Norton Healthcare, Norton Neuroscience Institute, Louisville, USA; 2 Neurological Surgery, University of Louisville School of Medicine, Louisville, USA

**Keywords:** allograft, cranioplasty, polyetheretherketone, allergic reaction, neurosurgery

## Abstract

Well-described complications of polyetheretherketone (PEEK) cranioplasty in pediatric patients include surgical site infection, post-operative hematoma, cerebral edema, and implant fracture. We present a rare case of hypersensitivity to PEEK presenting as an epidural effusion in a 7-year-old male receiving a PEEK cranioplasty following a decompressive craniectomy. Within three weeks, the patient experienced fever and emesis. Erythrocyte sedimentation rate (ESR) was high (>130 mm/Hr) as well as C-reactive protein (CRP) (6.4 mg/dL). A brain MRI with contrast demonstrated both subgaleal and epidural fluid collections with T2 isointense columns projecting from the galeal surface, through the holes in the implant to the dural surface. The patient appeared clinically well. A sterile tap of the pericranial fluid showed no growth, b2-transferrin was negative, but the IgG level was high (>129.2 mg/dL) in the tap fluid. High-dose steroids reduced the epidural collection, but then the collection returned with steroid wean. A second cranioplasty operation replaced the PEEK flap with autologous bone. Postoperative imaging demonstrated markedly reduced fluid collections and a decreased midline shift. The patient remained clinically intact throughout the experience. PEEK allergy following cranioplasty is a rare entity and must be distinguished from infection or hematoma. Medical treatment with steroids can be attempted, but, if refractory, then appropriate treatment may necessitate removal of the offending PEEK implant.

## Introduction

The incidence of cranioplasty failure ranges between 4%-25% [1], and complications may occur such as surgical site infection, incorrectly fitting implants, bone flap resorption, epidural and subdural hematomas, cerebral edema, and implant breaks [2,3]. In pediatric patients, the likelihood of bone resorption can be as high as 50% and is linked to younger age and the interval between craniectomy and cranioplasty [4-6]. Due to this complication, we have started offering allograft for the initial cranioplasty, usually with a custom-fabricated polyetheretherketone (PEEK) bone flap (KLS-Martin, Jacksonville, FL, USA).

Initially used for cranioplasty in 2007, PEEK has a number of advantages over both autologous and allograft options. PEEK is an aromatic, thermoplastic, and semi-crystalline polymer with ether and ketone chains [7-9]. Its material properties allow for CT-guided 3D printed customizable flaps. There have been no reported resorptions. The flap appears to promote natural bone remodeling along the borders, allowing for more natural growth patterns. PEEK has energy-absorbing properties similar to native bone, providing excellent cranial protection with a low rate of traumatic fragmentation. It has been reported that patients with PEEK implants during cranioplasty have lower rates of overall complications, implant failure (defined as implant infection or bone flap resorption requiring removal and/or replacement of implanted material), and infection compared to autologous bone or titanium mesh [10].

Implanting allograft material runs the risk of provoking an allergic reaction to alloplastic materials (PEEK, polymethylmethacrylate (PMMA), titanium mesh, hydroxyapatite) [11-14]. Allergic adverse events are exceedingly rare following PEEK implants [3,7,15]. Only one previous case has been reported after a PEEK cranioplasty [3]. In the current report, we present the rare case of an allergic response to a PEEK allograft plate in a pediatric patient. We will highlight the radiographic, diagnostic, and management issues and discuss the mechanism of the PEEK allergic reaction. 

## Case presentation

History and cranial surgery

A 7-year-old boy of Indian descent underwent a decompressive craniectomy for medically refractory intracranial hypertension resulting from a ruptured right frontal grade 2 arteriovenous malformation (AVM) by the Spetzler-Martin grading system. Two months later, he had a cerebral angiogram with embolization of the AVM, followed two weeks later by an open resection of the embolized AVM with placement of a custom PEEK flap. A detailed discussion with the family regarding the risks and benefits of an autologous versus allograft cranioplasty was conducted, and informed consent was obtained for the PEEK cranioplasty. An intraoperative angiogram confirmed an adequate resection of the AVM. There were no complications, and the patient was discharged on post-operative day 2.

Neurosurgical course following PEEK cranioplasty

Within three weeks of the PEEK cranioplasty, the patient began experiencing a fever and had a single episode of emesis. Evaluation revealed an intact neurological exam. The laboratory investigation is presented in Table 1.

**Table 1:** Laboratory investigation following PEEK cranioplasty PEEK: polyetheretherketone; WBC: white blood cells; ESR: erythrocyte sedimentation rate; CRP: C-reactive protein; IgG: Immunoglobulin G.

Laboratory Test	Laboratory Value	Reference Range
Within 3 week of PEEK cranioplasty:		
WBC	9.81 K/uL	5-14.5 K/uL
ESR	>130 mm/Hr	0-22 mm/Hr
CRP	6.9 mg/dL	<1.0 mg/dL
Within 3 months of PEEK cranioplasty:		
ESR	51 mm/Hr	0-22 mm/Hr
CRP	4.2 mg/dL	<1.0 mg/dL
4 months after PEEK cranioplasty:		
IgG	>129.2 mg/dL	0-3.3 mg/dL

**Figure 1:**
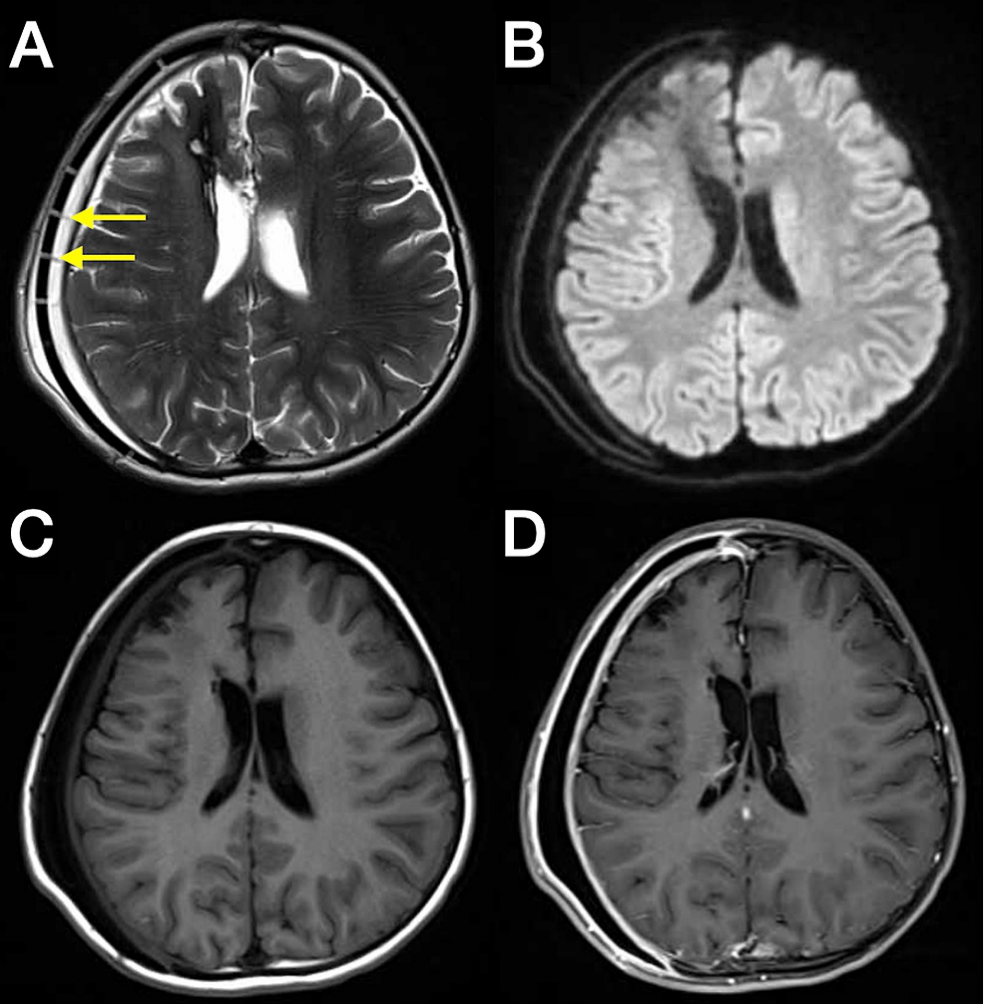
Brain MRI with and without gadolinium contrast three weeks after the PEEK cranioplasty Brain MRI with and without gadolinium contrast three weeks after the polyetheretherketone (PEEK) cranioplasty demonstrated both epidural as well as subgaleal fluid collections. (A) Isointense columnar structures extending from the inner surface of the galea to the epidural surface (arrows) within hyperintense fluid signal; (B) diffusion weighted sequence with hypointense subgaleal and epidural fluid signal, inconsistent with infection; (C) pre- and (D) post-contrast T1 with smooth, homogeneous enhancement of the dura and galea, felt to be more consistent with reactive than infectious etiology.

A brain MRI with and without gadolinium contrast demonstrated a small epidural collection subjacent to the cranioplasty graft (8.5 mm in maximum length) as well as a T2 hyperintense collection overlying the graft (5.7 mm in maximum width) (Figures 1A-1D). 

There was no midline shift but of significant note were isointense columns within the epidural effusion which appeared to emanate from the inner surface of the galea, project through the manufactured holes in the flap, and reach the epidural surface. The flap was well aligned to the native skull, and the patient appeared clinically well. He was admitted for an infectious work-up which was negative. We felt his imaging was unusual but did not require revision or surgical intervention. The patient was discharged on day 2 with his C-reactive protein (CRP) and erythrocyte sedimentation rate (ESR) trending down, and there were no fevers for 24 hours.

**Figure 2:**
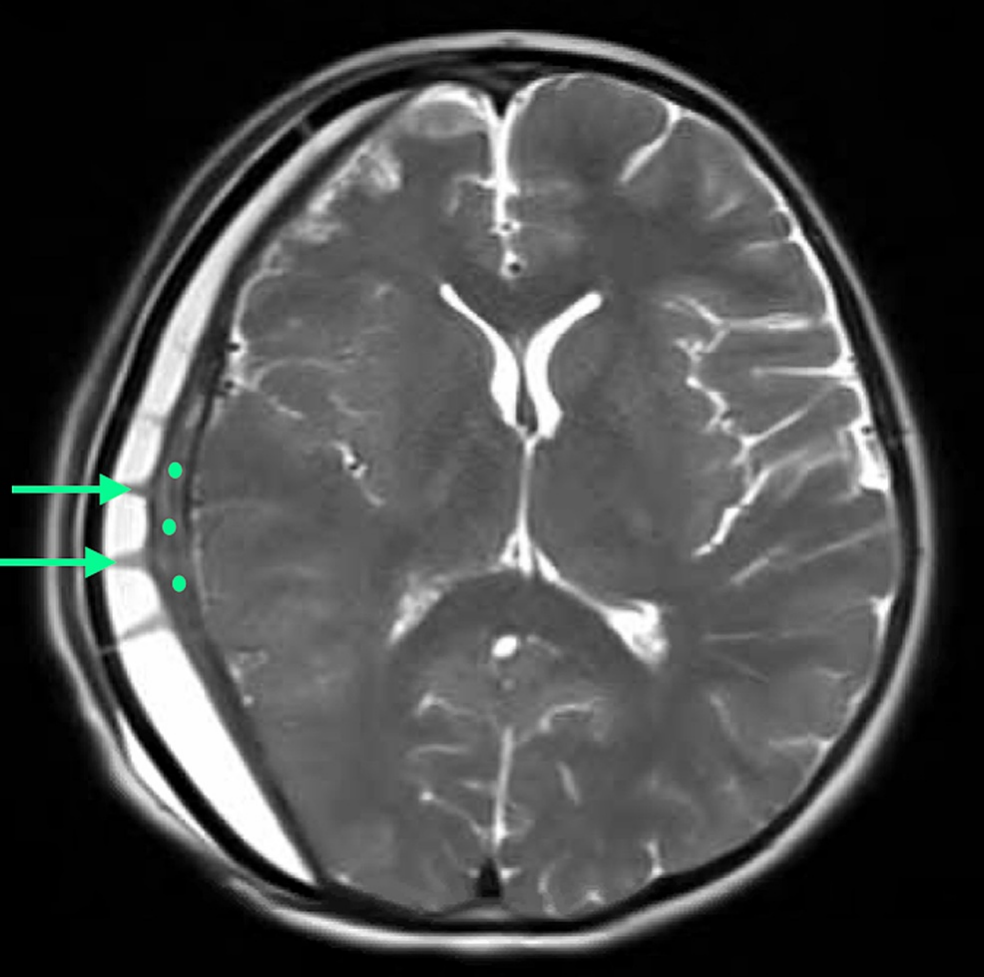
Brain MRI three months after the PEEK cranioplasty Limited brain MRI three months after the polyetheretherketone (PEEK) cranioplasty revealed a larger homogeneous epidural collection subjacent to the cranioplasty, with prominent columnar isointense structures (arrows) and a relatively thick and slightly fluctuant-appearing isointense layer on the epidural surface (dots).

Three months after his cranioplasty, the patient returned with an increasing subgaleal fluid collection. He received a limited brain MRI, revealing a larger heterogeneous epidural collection subjacent to the cranioplasty with prominent linear isointense structures and an isointense and slightly fluctuant-appearing collection overlying the dura (Figure 2). 

There was both a midline shift and effacement of the right hemisphere. Both the ESR rate and CRP had declined (as seen in Table 1), and he remained clinically stable with the exception of transient ipsilateral eye pain, deemed non-ocular in etiology by ophthalmology. Because of his clinical status, conservative management was continued. The patient returned with posterior incisional thinning four months after the cranioplasty. A sterile tap of the pericranial fluid was obtained, showing no infectious growth, a negative b2-transferrin, but with a greatly elevated IgG level (Table 1). The decision to perform a tap of the pericranial fluid was based on worsening fluid collection with the pressure causing clear scalp thinning which put the cranioplasty under threat. Since the patient was not acting infected, we thought it was doubtful that antibiotics would be of benefit. Furthermore, a CSF leak was also unlikely since the fluid began to collect several weeks after the cranioplasty. Therefore, a tap was the lowest cost and highest yield way to determine the definitive diagnosis. 

**Figure 3:**
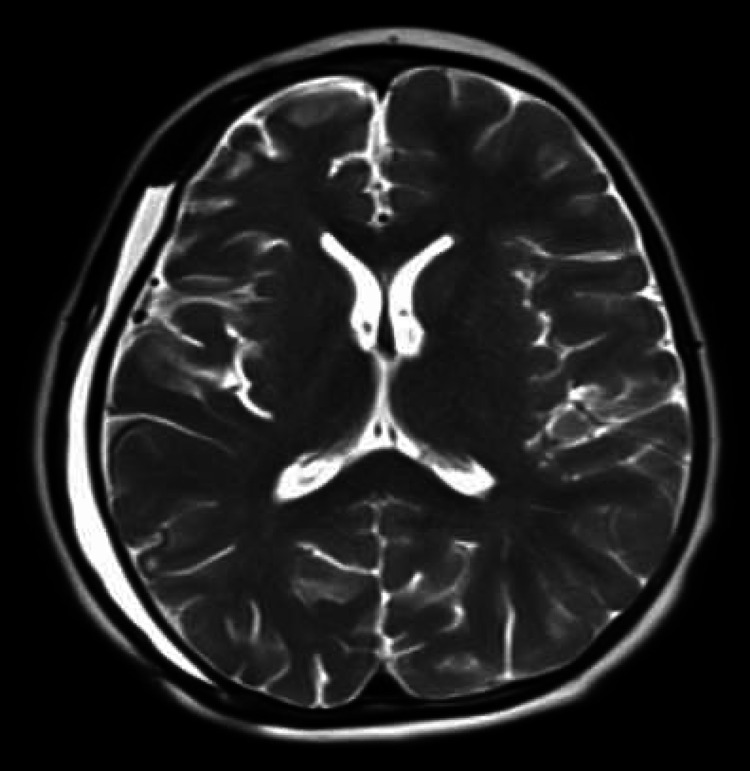
Brain MRI shunt series one month after removal of the PEEK cranioplasty Limited brain MRI shunt series one month after removal of the polyetheretherketone (PEEK) cranioplasty demonstrating resolution of the midline shift, right hemispheric compression, and columnar structures. The epidural collection is markedly reduced in size.

Given his likely allergy to PEEK, we initiated medical management with 2.5 mg of dexamethasone PO every six hours for one week, followed by a very slow taper over four weeks. This treatment initially proved effective, with moderate improvement of the right hemispheric compression and midline shift as well as complete resolution of both the isointense epidural effusion and subgaleal fluid. Within one week of the steroid taper, his symptoms had returned. We felt this improvement with steroids and relapse with taper was indicative and consistent with PEEK allergy. There was no clinical evidence for infection. After failing medical management, a revision cranioplasty with autologous flap was performed. Intraoperatively, the only significant finding was a friable and poorly vascular accretion on the dural surface that was easily removed. The collection was reminiscent of an infectious collection but without local tissue destruction, foul odor, or odd color. Because the patient had already had an infectious workup (including a tap of the subgaleal collection) and had improved with steroids, and then worsened off of steroids, no sample was sent to pathology for evaluation. One month following autologous replacement, the patient was doing well, with resolution of the posterior incisional thinning. A brain MRI showed resolution of the right hemispheric compression and isointense linear structures, with some residual epidural fluid (one-third of its previous volume) (Figure 3). 

## Discussion

On market allograft materials include titanium mesh, PMMA, PEEK, and bone cement. The benefits and disadvantages associated with these materials are depicted in Table 2 [2,9,10,16,17].

**Table 2:** Advantages and disadvantages of materials used in cranioplasty

Material	Advantages	Disadvantages
Autologous bone graft (bone flap replacement)	Most common practice	Risk of resorption and fragmentation
	No immune rejection	Requires freezer cryopreservation or subcutaneous abdominal implantation
	Lowest cost	Highest rates of infection (25.9%) compared with titanium mesh, PMMA, and alumina ceramics
	Good bony ingrowth	
	Perfect fit/good aesthetics	
Other bone/fascia materials	Readily available	Second surgical site
	No immune rejection	
	Low cost	Not common – little information published on risk of resorption and infection
	Good bony ingrowth	
Titanium mesh	Precise	Implant failure: exposure of metal plates/screws, requires additional surgery for removal (5.3%)
	Time-saving	High cost
	Lowest rate of graft infection of all cranioplasty materials (2.6%)	Artifacts can obscure subsequent CT/MRI
	Computer-assisted 3D modeling to design titanium mesh implants	
Polymethylmethacrylate (PMMA) (acrylic)	Low cost	Brittle, with risk of traumatic fragmentation which are difficult to detect
	Radiolucency	No bony ingrowth
	Durable	Risk of resorption
	Strong	High risk of infection (12.7%)
	Heat resistant	Residual monomer from cold polymerization may be toxic
	Inert	
Polyetheretherketone (PEEK) (plastic)	Resistance to gamma and electron beam radiation	High cost
	No resorption	
	Energy absorbing properties similar to bone: very low risk of traumatic fragmentation	
	3D computer-designed to fit cranial defect	
	Radiolucent and non-magnetic (no artifacts with imaging)	Lack osteointegrative properties
	High tensile strength (103 MPa)	
	Light material with low density	
	Same rate of infection as native bone	
Hydroxyapatite (HA) (bone cement or bioceramics)	Good scaffolding material for bony ingrowth	Brittle, risk of traumatic fragmentation
	Low postoperative infection rate (5.9%)	Time consuming, needs to be extensively contoured intra-operatively
	Chemically stable	Difficult to apply over large areas
	Comparable tissue compatibility to acrylics	Very expensive

Initially developed for aerospace purposes in 1978, PEEK has emerged as a valuable component in healthcare, utilized in spinal, dental, maxillofacial, cardiovascular, and cranioplasty procedures [9]. While PEEK implants offer a plethora of advantages in cranioplasties, complications may ensue postoperatively (Table 2). In Morselli and colleagues’ study of 1688 patients who underwent implantation of custom-made prostheses, 348 complications (20.64%) were reported including 49 involving PEEK [8]. Infections due to PEEK were the most common adverse events, followed by epidural hematomas, hydrocephalus, fluid collection, and graft displacement. No allergic responses were documented. In Jonkergouw and colleagues’ study of 40 cranial PEEK implants in 38 patients, the overall complication rate was 28%, with infections comprising the highest percentage followed by a postoperative hematoma and CSF leak [16]. In Punchak and colleagues’ study of 183 patients who underwent cranioplasties with PEEK, complications developed postoperatively in 28 patients (15.3%), with implant failure and infections most common [10]. In all three studies involving PEEK as a component of the cranioplasty, no PEEK allergies were reported. 

Only three cases of allergic reactions to PEEK implants have been reported in the literature, specifically, an intervertebral PEEK cage implanted during an anterior cervical discectomy and fusion (ACDF) [15], a PEEK-containing device implanted during a rotator cuff repair [7], and a PEEK implant placed during a bilateral cranioplasty (Table 3) [3].

**Table 3:** Allergic reactions to polyetheretherketone (PEEK) implant IgG: Immunoglobulin G; ESR: erythrocyte sedimentation rate; CSF: cerebrospinal fluid.

Study	Surgical procedure	Symptoms and duration after implantation	Radiological/Laboratory Findings	Treatment	Outcomes
Maldonado-Naranjo et al. [15]	Intervertebral PEEK cage implanted during ACDF	Four weeks after implantation: generalized weakness, fatigue, diffuse erythema and pruritus most of body, tongue swelling, throat redness, bilateral eye swelling	Skin patch testing with PEEK: severe erythema and blistering	Removal of PEEK intervertebral cage	Improved symptoms within hours of implant removal
Kofler et al. [7]	PEEK-containing device implant after rotator cuff injury	Eight hours after implantation: pain and erythema at surgical site	Negative for bacterial/fungal infection	Removal of rotator cuff device	Resolution of symptoms
			Device head containing PEEK was implanted in an abdominal pouch: perifocal edema 8 hours later; histology revealed panniculitis with leucocytic infiltration		
Qiu et al. [3]	Bilateral craniectomy after TBI; PEEK implant in bilateral cranioplasty	Seven days after implantation: headache	Head CT: epidural effusion	Subcutaneous drainage	Postoperative CT: effusion resolved
			Elevated IgG (52 mg/dL)	Dexamethasone 10 mg/day	
			Normal glucose	PEEK implant not removed	Symptoms improved
			Negative for bacterial infection		
Current Study 2021	Cranioplasty with PEEK allograft plate after decompressive craniectomy for ruptured AVM	Three weeks after implantation: fever, emesis	Brain MRI with contrast: epidural and subgaleal fluid collection	Dexamethasone 2.5 mg	Brain MRI 1 month after PEEK removal: resolving fluid collections, decreased midline shift
			ESR: >130 mm/Hr (0-20 mm/Hr)		
			C-reactive protein: 6.4 (<1.0 mg/dL)	Replaced PEEK flap with patient’s autologous bone	Symptoms resolved
			CSF culture: no growth, b-transferrin negative, IgG >129.2 mg/dL (0-3.3 mg/dL)		

The initiation of symptoms ranged between eight hours and four weeks after PEEK implantation. Confirmation of the PEEK allergy included either skin patch testing with PEEK, the PEEK device implanted in the abdomen, and laboratory findings negative for bacterial/fungal infections and with a high IgG. The PEEK implants were removed in the patients who underwent an ACDF and rotator cuff repair with subsequent resolution of their symptoms. Only one patient in the literature, a 25-year-old man of Chinese ethnicity, experienced a PEEK allergic reaction following cranioplasty, exhibited by a headache seven days after implantation [3]. A head CT confirmed an epidural effusion, the glucose level was normal, the IgG level was elevated (52 mg/dL), and bacteriological results were negative. Following subcutaneous drainage and dexamethasone 10 mg/day, the patient’s symptoms improved and the effusion resolved as confirmed by CT. The PEEK implant was not removed. 

The mechanism of the allergic reaction to PEEK was discussed in two of these previous cases. Kofler and colleagues reported that the patient who underwent the PEEK-containing device implant during rotator cuff repair had a previously known, severe type IV allergy to epoxy resin [7]. These authors described that PEEK synthesis was based on dialkylation of bisphenolate salts and that epoxy resin is mainly based on bisphenols. They observed a local inflammatory reaction after device implantation, although epicutaneous testing was negative. These findings may be due to increased exposure of the PEEK device to mediator cells in the subcutaneous tissue. Qiu and colleagues surmised that a non-specific immune response to the surgical implant as reflected by the elevated IgG level was the cause of the PEEK allergy following cranioplasty [3]. Furthermore, previous studies reported the association between implant-related allergic reactions and type IV delayed-type hypersensitivity [18-20]. These particular hypersensitivities may occur a few days to several years after contact with the allergens [18]. 

Such distinct radiographic findings as presented in our case have not previously been reported. The allergic reaction created distinct, linear, and isointense radial columns apparent on MRI, projecting through the manufactured holes in the flap. Type IV reactions require the recruitment of phagocytes and lymphocytes, both mediated by cytokines and emanating from the scalp. It is perhaps the case that the MRI shows the path of least resistance to this process, and the resulting granulomatous organization of these cells forms columns through the manufactured holes in the flap.

This is the first report of a pediatric patient who experienced a PEEK allergy following a cranioplasty, requiring surgical removal rather than steroid treatment alone. The most common complications following cranioplasty are infections, hematomas, hydrocephalus, and implant failure. Implant allergy is exceptionally rare and therefore, an index of suspicion is required for diagnosis. Our patient was clinically well throughout his course, except for a brief fever, one episode of emesis, and some transient ipsilateral eye pain. While our early suspicion based on MRI was either infection, CSF hygroma, or hydrocephalus, his clinical condition made infection unlikely. A tap of the fluid confirmed this with a negative culture, and a negative b2-transferrin ruled out a CSF origin. IgG levels were markedly elevated, and the effusion improved with steroids. After steroid weaning, the symptoms worsened again, and subsequent replacement of the PEEK flap with the patient’s autologous bone brought near-complete resolution. The mechanism was most likely a type IV hypersensitivity to the PEEK implant.

## Conclusions

Neurosurgeons should be aware of a rare allergic response which can become a complication of a PEEK cranioplasty, and a high index of suspicion is required. Infection and hematoma should be ruled out first. A thorough history, comprehensive laboratory analysis focusing on allergy-specific findings, and cranial imaging studies may shed light on the correct etiology. The unique radiographic appearance, lack of infectious markers, and improvement with steroids were suggestive of allergic reaction in this case. Further investigation is warranted into the mechanisms associated with autoimmune reactions to a PEEK cranioplasty. 
